# Role of Splice Variants of Gtf2i, a Transcription Factor Localizing at Postsynaptic Sites, and Its Relation to Neuropsychiatric Diseases

**DOI:** 10.3390/ijms18020411

**Published:** 2017-02-15

**Authors:** Yoshinori Shirai, Weidong Li, Tatsuo Suzuki

**Affiliations:** 1 Department of Neuroplasticity, Institute of Pathogenesis and Disease Prevention, Shinshu University Graduate School of Medicine, 3-1-1 Asahi, Matsumoto 390-8621, Japan; yoshirai@shinshu-u.ac.jp; 2 Bio-X Institutes, Key Laboratory for the Genetics of Development and Neuropsychiatric Disorders (Ministry of Education), Shanghai Key Laboratory of Psychotic Disorders, and Brain Science and Technology Research Center, Shanghai Jiao Tong University, 800 Dongchuan Road, Shanghai 200240, China; liwd@sjtu.edu.cn; 3 Institute for Biomedical Sciences, Interdisciplinary Cluster for Cutting Edge Research, Shinshu University, 3-1-1 Asahi, Matsumoto 390-8621, Japan; 4 Department of Neuroplasticity, Institute of Pathogenesis and Disease Prevention, Shinshu University Graduate School of Medicine, 3-1-1 Asahi, Matsumoto 390-8621, Japan; 5 Department of Biological Sciences for Intractable Neurological Diseases, Institute for Biomedical Sciences, Interdisciplinary Cluster for Cutting Edge Research, Shinshu University, 3-1-1 Asahi, Matsumoto 390-8621, Japan

**Keywords:** postsynaptic density, synapse, local protein synthesis, transcription factor, neuropsychiatric disorders, 5′UTR, alternative splicing, synaptic plasticity

## Abstract

We previously reported that various mRNAs were associated with postsynaptic density (PSD) purified from rat forebrain. Among the thousands of PSD-associated mRNAs, we highlight the biology of the general transcription factor II-I (*Gtf2i*) mRNA, focusing on the significance of its versatile splicing for targeting its own mRNA into dendrites, regulation of translation, and the effects of *Gtf2i* expression level as well as its relationship with neuropsychiatric disorders.

## 1. Introduction

We previously reported that a large number of mRNAs are associated with postsynaptic density (PSD) prepared from rat forebrain [1,2]. Later, deep sequencing of hippocampal neuropil-residing mRNAs supported the abundance of RNAs in dendrites/axons [3]. Among the thousands of PSD-associated mRNAs, more than 100 mRNAs encode nuclear proteins.

De novo synthesis of proteins is necessary for the induction of plastic modification in stimulated synapses, which form the molecular basis of learning and memory [4,5]. Translation of postsynaptically localized mRNAs at the stimulated synapses is one of the mechanisms for spatiotemporally specific modifications in stimulated synapses [6,7]. Short-term plastic modifications in synapses are explained by modification and mobilization into synapse active zones of synaptic proteins, such as receptors, cytoskeletons, and various signaling molecules. However, this may not be enough for the long-term or long-lasting maintenance of the plastic modification in synapses. Long-lasting synaptic modification (maintenance and stabilization) requires novel protein synthesis both at postsynaptic sites and via transcription in the nucleus [8]. For example, transcription of brain-derived neurotrophic factor (BDNF) [9] and Arc [10,11] is induced after neural activation. Synapse-to-nucleus signal transmission should occur ahead of the transcription of these genes.

**Figure 1:**
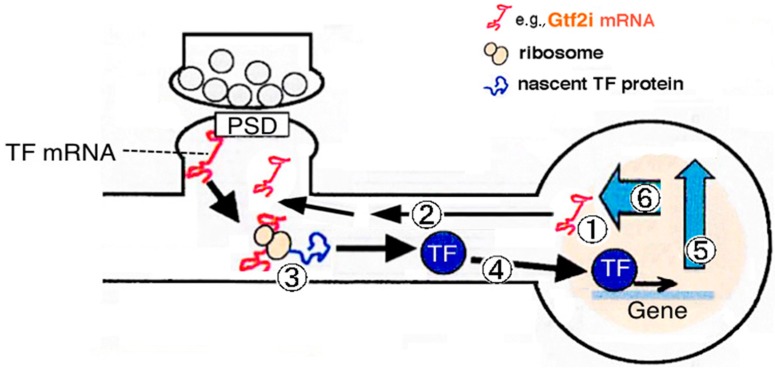
Transcription, targeting, and the fate of postsynaptically localizing mRNAs that encode transcription factors. The mRNA encoding Gtf2i is one of the postsynaptic density (PSD)-associated mRNAs. It is hypothesized that locally translated transcription factors at the postsynapse act as signal transduction molecules transmitting synaptic signals into the nucleus. 1. Transcription of its own gene; 2. Targeting mRNA into the dendrite; 3. Postsynaptic local translation; 4. Synapse-to-nucleus transmission of protein products; 5. Modulated transcription of downstream genes in the nucleus; 6. Feedback effects on synapse (Multiple processes/pathways are plausible; however, their details are still unknown). TF: transcription factor.

Dendritic mRNAs encoding nuclear proteins contained mRNAs encoding transcription factors. These transcription factor-encoding mRNAs are supposed to be transferred into the neuronal dendrites and locally translated there upon synaptic stimulation. Their products are translocated back into the nucleus, where they transcribe downstream genes involved in long-term synaptic modification (Figure 1). Maturation of mRNAs may occur in the dendrites, in addition to cell soma [12]. The mRNA encoding general transcription factor II-I (Gtf2i) is one of these PSD-associated mRNAs. We hypothesize that postsynaptic localization of *Gtf2i* mRNA is related to two events—local translation at the postsynapse and synapse-to-nucleus signal transduction. Locally translated transcription factors at the postsynapse act as signal transduction molecules transmitting synaptic signals into the nucleus. The localization of mRNA encoding a transcription factor at the postsynaptic sites is studied for the first time in *Gtf2i*. Therefore, until now, there has been no report revealing the fate of this protein at the postsynaptic site and its role in synaptic function. However, our hypothesis is also based on other transcription factors and molecules, such as cAMP responsive element binding protein (CREB), signal transducer and activator of transcription 3 (STAT3), Mothers against dpp (SMAD), huntingtin, and importin, that translocate from postsynaptic sites to the nucleus [7,13,14,15,16,17,18].

In this review, we would discuss the regulation of the Gtf2i expression in the postsynaptic area, its downstream cellular events, and the association of *GTF2I* with neuropsychiatric disorders.

## 2. Role of Splice Variation in 5′ Untranslated Regions of *Gtf2i*

### 2.1. Role of 5′ Untranslated Region Variations in Gtf2i mRNA for Regulation of Its Own Transcription

Transcriptional regulation is achieved by promoters localizing upstream of each transcription start site (TSS). A promoter is activated by its specific intrinsic transcription factors in each of the 5′ untranslated regions (5′UTR). Alternative promoters of *Gtf2i* have been identified in humans and mice. In human *GTF2I*, three additional exons between exon 1 (encoding the known 5′UTR) and exon 2 (encoding the start codon of *GTF2I*), three TSSs, and four alternative 5′UTRs have been identified [19]. In mouse *Gtf2i*, two additional exons between exon 1 and exon 2, three TSSs, and five alternative 5′UTRs have been identified [19]. In rats, we identified seven novel TSSs, six novel exons between the exon 1 and the exon 2, and eight transcripts (including seven novel transcripts) of *Gtf2i* with different 5′UTRs [20]. Upstream of the TSSs of all of the *Gtf2i* 5′UTRs identified so far, each *Gtf2i* 5′UTR has its own intrinsic transcription factor-binding sites [19,20]. Multiple promoters of *Gtf2i* make transcription responsive to various stimuli in different types of cells. Multiple promoters of *Gtf2i* also enable production of various splice variants with different 5′UTRs, and multiple 5′UTRs make *Gtf2i* mRNA translation responsive to various stimuli at different cellular and subcellular locations.

Another possible mechanism for control of transcription of the numerous *Gtf2i* variants themselves is epigenetic control, since the novel 5′UTRs that we identified are embedded within CpG islands [20], where methylated cytosines could be involved in transcriptional repression. Neural activity decreases CpG methylation and activates transcription of target genes via release of repressor proteins [21,22]. This mechanism may enable cell type-specific and/or neural activity-dependent expression of each 5′UTR variant.

### 2.2. Role of 5′UTR Variation in Gtf2i mRNA for Subcellular Localization

Various mRNAs localize to dendrites in neuronal cells and the 5′UTR and/or 3′UTR of mRNA plays important roles in the localization to as well as local translation in dendrites [6]. The 5′UTR and the 3′UTR of mRNA play important roles in its subcellular localization, translation regulation, and stability [6,7,23,24]. This appears to be the case for *Gtf2i*. *Gtf2i* mRNAs with different 5′UTRs showed differential expression patterns in rat brains. Among eight isoforms, one was detected in dendritic processes of neuronal cells, suggesting that dendritic localization of *Gtf2i* mRNA variants is dependent on 5′UTR variation [20]. This variant may play a synapse-specific role, while other variants may play non-synaptic roles.

### 2.3. Regulation of Translation and Localization of mRNA by RNA-Binding Proteins Interacting with 5′UTRs

The presence of two *cis*-acting factors, G-quadruplex [20] and stem-loop structure (our unpublished data), is predicted in the 5′UTR of *Gtf2i* mRNA variant localizing to neuronal dendrites. The G-quadruplex is a guanine (G)-rich nucleic acid sequence that forms a four-stranded structure. The G-quadruplexes in the 5′UTRs of mRNAs play important roles in the localization of mRNAs to neuronal dendrites [25] and translation regulation of many mRNAs localized postsynaptically [26]. Some RNA-binding proteins (RBPs), as *trans*-acting factors, regulate translation, subcellular localization, and metabolism of mRNAs via binding to UTRs (both 5′UTRs and 3′UTRs) [27]. A well-known RBP for G-quadruplexes is fragile X mental retardation protein (FMRP), which is localized to dendrites, binds to the 5′UTR and/or 3′UTR of target mRNAs, and negatively regulates translation of the target proteins in dendrites [28,29,30,31]. Thus, FMRP negatively regulates translation of target mRNAs via direct binding to the G-quadruplex structure in 5′UTR[31]. FMRP, via its activity in dendrites, is related to the formation and maintenance of synapse structure and spine shape [32]. Abnormality in FMRP is linked to autistic symptoms of the fragile X syndrome.

The stem-loop structure of mRNA in the 5′UTR is important for translational regulation. One RBP known to interact with this structure is RNA helicase A, which recognizes this structure in the 5′UTR of various mRNAs, unwinds secondary structures, and facilitates ribosome association to enable efficient cap-dependent translation [33,34].

Thus, a mechanism involving specific RBPs is likely to regulate localization and translation of *Gtf2i* mRNA variants. Identification and functional analysis of RBPs that specifically recognize RNA structures in the dendritic 5′UTR of *Gtf2i* mRNA is an important next step. Our recent study identified RNA helicase A as one of RBPs that specifically bind to the dendritic 5′UTR of *Gtf2i* mRNA [35] although the site of interaction has not yet been identified. This study failed to detect FMRP until now. For its detection, standardization of the binding conditions for preservation of the optimum G-quadruplex structure may be required.

### 2.4. 5′UTR Works as a “Spatial Code” and a “Quantitative Code”

*BDNF* is an extensively studied example of a gene, similar to *Gtf2i*, with multiple splice variants with different 5′UTRs. Analogy with *BDNF* may give us a hint about the functional significance of multiple variations in *Gtf2i* 5′UTR. Human *BDNF* has 34 alternative mRNAs, which are combinations of a single coding region, 17 different 5′UTRs, and 2 different 3′UTRs with different polyadenylation sites [36]. Rodent *Bdnf* has 22 alternatively spliced mRNAs, which are combinations of a single coding region, 11 different 5′UTRs (I, IIA, IIB, IIC, III, IV, V, VI, VII, VIII, and IXa), and 2 different 3′UTRs with different polyadenylation sites [37]. Systematic analysis of subcellular localization of each 5′UTR in cultured rat hippocampal neurons suggests that alternatively spliced 5′UTRs selectively determine the intracellular localization of *Bdnf* mRNA variants to the soma, proximal dendrites (I, IV), or distal dendrites (IIC, VI) [38]. Furthermore, targeting of *Bdnf* mRNA variants into distal dendrites is controlled by neural activity [9]. In untreated rat hippocampal tissues, dendritic enrichment of 5′UTR variants (VI, VII in CA1; I, VI, IXa in CA3; V, VI, VII, VIII in DG) was observed. Upon neural stimulation, levels of *Bdnf* transcripts in dendrites were upregulated or downregulated, depending on variant types and brain region (e.g., in the CA1 region, variant mRNAs II, IV, and VI were upregulated, while variant mRNA III was downregulated) [39].

Based on these results, the “spatial code hypothesis” suggesting that different 5′UTRs regulate intracellular localization of the corresponding mRNA variants was proposed [38]. This “spatial code hypothesis” may also be applicable to *Gtf2i* mRNAs and could be generalized, since we found selective localization of certain type of *Gtf2i* mRNA variants to dendrite [20], although there are no distinctive motifs or sequences conserved between BDNF mRNA and Gtf2i mRNA.

Another functional feature of alternative 5′UTR of *Bdnf* is translation regulation. Translatability of each 5′UTR was assessed by an in vitro luciferase assay in a neuroblastoma cell culture model. *Bdnf* mRNAs with different 5′UTRs are translated differently in the basal state, and their translations were modulated differently with synaptic stimulations, such as KCl, BDNF, AMPA, NMDA, dopamine, or 5-HT [40]. Based on this, the “quantitative code hypothesis” was proposed, where translatability of *Bdnf* mRNA variants, and hence the expression level of BDNF protein, is determined by each 5′UTR in response to specific stimuli [40]. In this hypothesis, the factor that determines the expression level is intrinsic to the 5′UTR. Translatability of the variant mRNAs is an important factor critically affecting the expression level of protein. Therefore, 5′UTR bears a “quantitative code.” This “quantitative code hypothesis” may also be applicable to *Gtf2i* mRNA and could be generalized, since *Gtf2i* mRNA also has multiple 5′UTR variants with structures that affect translation efficiency.

## 3. Downstream of *Gtf2i* Local Translation

### 3.1. Synapse-to-Nucleus Signal Transduction via Gtf2i

Gtf2i is a signal-induced transcription factor, responding to signals, such as growth factor stimulation, TGFβ signaling, ER stress signaling, calcium signaling, immune signaling, and c-Src-dependent transcription activation [41,42,43,44]. Thus, this transcription factor is a versatile regulator of numerous cellular processes.

Transcription is induced by neural activity [8]. Several transcription factors, such as CREB, STAT3, and SMAD, are localized away from the soma at distal axons, locally synthesized upon stimulation, and translocated to the nucleus [7,13,14,15]. To our knowledge, there are no reports on locally translated transcription factors at the postsynaptic site. *Gtf2i* mRNA, at least one type of mRNA splice variant (rDEC4ED sequence-containing mRNA, see [20]), is localized at postsynaptic sites and is likely to be translated in a synaptic stimulation-dependent manner at the synapses. Gtf2i synthesized at postsynaptic sites may translocate into the nucleus, via nuclear localization signals (NLSs), and regulate expression of their downstream target genes. Gtf2i variants play differential roles in signal-induced transcription regulation. For example, the β isoform represses transcription of c-Fos, while the Δ isoform acts as an activator in murine fibroblasts [42].

### 3.2. Downstream Target Genes and Cellular Functions of Gtf2i

Various downstream genes and cellular functions of Gtf2i have been unveiled. In a promoter binding study, downstream target genes for Gtf2i transcription factor included those involved in axon guidance, neurodevelopmental disorders, calcium signaling, and the cell cycle [45]. Though the knockout in the mouse is embryonically lethal, *Gtf2i^−/−^* mouse embryos showed alterations in gene expression related to TgfbrII/Alk1/Smad5 and Vegfr-2 signaling cascades [46], suggesting that Gtf2i is upstream of these signaling pathways during embryogenesis.

Involvement of Gtf2i in the transcription of the *Dlx5/Dlx6* homeobox genes has been suggested [47]. Gtf2i binds to the I56i enhancer region of the *Dlx5/Dlx6* and possibly modulates affinity of Dlx1 and Dlx2 towards the I56i enhancer region [48] to regulate transcription of *Dlx5/Dlx6* [47]. This suggests that Gtf2i is involved in the maturation of inhibitory interneurons since Dlx5 and Dlx6 are involved in the differentiation and migration of GABA-expressing interneurons in the forebrain [47,49]. Thus, Gtf2i is proposed to be a regulator of *Dlx5/Dlx6* expression via activation of the I56i enhancer region and may regulate maturation of GABAergic interneurons and finally alter excitatory/inhibitory balance of neural circuits.

Additionally, GTF2I has been found to regulate transcription of *DYX1C1* via binding to the promoter (or 5′UTR) region of *DYX1C1* [50]. *DYX1C1* is implicated in dyslexia [51], neural migration [52], cortical development, and spatial learning [53,54]. Thus, abnormality of GTF2I is related to the pathogenesis of dyslexia possibly via impaired *DYX1C1* transcription [50]. These reports suggest that Gtf2i is an upstream regulator of various brain functions, including neuronal development, inhibitory synapse maturation, and neural circuit formation.

### 3.3. Coding Variants May Differentially Regulate Downstream Transcription and Cellular Processes

Combination of four in-frame cassettes of exons 9–12 produces Gtf2i variants in the coding region, and nine such coding variants have been discovered—Gtf2iα (exons 9–10–11), Gtf2iβ (exons 9–11–12), Gtf2iγ (exons 9–10–11–12), Gtf2iΔ (exons 9–11), Gtf2iε (exons 11–12), Gtf2i(9–12), Gtf2i(9), Gtf2i(12), and Gtf2i(−) [19,20]. The Gtf2i protein has an N-terminus leucine zipper, six I-repeat DNA-binding motifs, and two NLSs [44]. The presence and number of these motifs are not changed among these coding variants. Variations in combinations of exons 9–12 may affect complex conformation of Gtf2i proteins [55] and different combinations of Gtf2i variant dimers regulate target genes [42,56]. Each splice variant in the coding region of Gtf2i is possibly involved in the transcription of its own set of target genes [55,56]. Thus, Gtf2i variants in the coding region may be involved in the differential regulation of the expression of target genes via variable Gtf2i dimer organization (see review by Roy [44]).

In addition, variations in the isoforms of the coding region with different combinations of exons 9–12 change the length of the PEST sequence (a protein sequence enriched in proline, glutamate, serine, and threonine), which may lead to changes in the stability of this protein [20]. Furthermore, there are specific combinations of the variants in the 5′UTRs and those in the coding region [20]. Therefore, the properties and functions of Gtf2i are also affected by the corresponding 5′UTR, which regulate localization of Gtf2i mRNA. This suggests that Gtf2i function varies depending on its cellular and subcellular localization.

Phosphorylation of the tyrosine^248^ residue, which is encoded by exon 9, is involved in translocation to the nucleus and activation of the transcriptional activity of Gtf2i [41,57]. Isoforms lacking exon 9, Gtf2i(12), and Gtf2i(−) in the brain may have functions other than transcriptional regulation in the nucleus. Another function of Gtf2i outside the nucleus is agonist-induced calcium entry, as has been suggested for the Δ isoform [58]. However, this function of Gtf2i may not occur in the brain Gtf2i since the Δ isoform is not expressed in the brain tissue.

## 4. Relationship between *GTF2I* and Neuropsychiatric Abnormality

### 4.1. Copy Number Variation in Williams–Beuren Syndrome Region and Neuropsychiatric Diseases

The human *GTF2I* is implicated in Williams–Beuren syndrome (WBS) and 7q microduplication syndrome (7dupASD; ASD, autism spectrum disorder). WBS is characterized by altered facial appearance, cardiovascular abnormalities, severe visuospatial cognitive abnormalities, and abnormal social behavior (hypersociability) [59,60]. The linkage of the *GTF2I* gene to WBS was suggested by the fact that patients with WBS exhibit hemizygous deletion of a 1.5 Mb region in chromosome 7q11.23 (WBS region), containing 28 genes, including *GTF2I* [59,60]. Thus, *GTF2I* is regarded as one of genes responsible for human WBS [60,61]. Genotype–phenotype correlation studies of patients with mild WBS phenotype and normal IQ carrying a shorter deletion of this region have shown that *GTF2I* contributes to the alteration in social behavior of patients with WBS, but not to visual-spatial cognitive impairment, craniofacial features, and other WBS features [59,61]. The linkage between *GTF2I* and WBS is also supported by a study on haploinsufficiency of *Gtf2i* mice. *Gtf2i* heterozygous mice showed increased social interaction, similar to the behavior in patients with WBS, although learning and memory, especially spatial memory, were normal in these mice [62].

**Figure 2:**
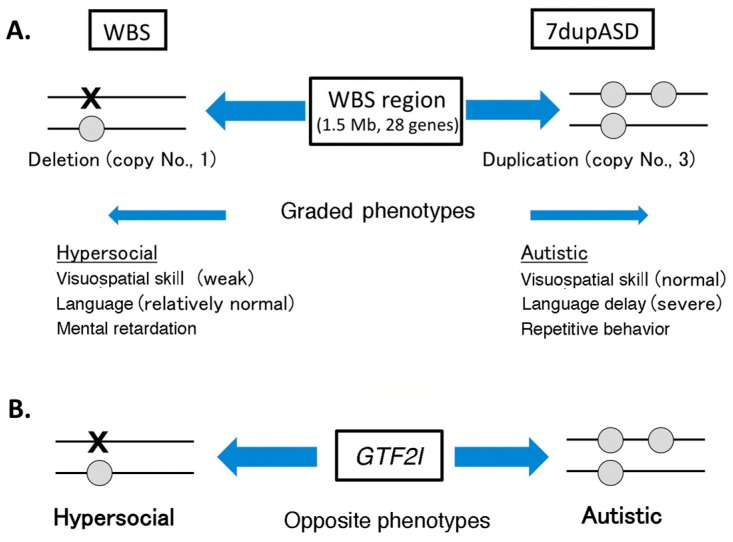
Psychiatric phenotypes dependent on copy number variations (CNV) of the Williams–Beuren syndrome (WBS) region and *GTF2I*. (**A**) Effects of CNV on the WBS region; (**B**) Effects of CNV on *GTF2I*. Characteristic features of WBS and 7dupASD are listed. Gray circles indicate presence of a single copy of the *GTF2I* and X indicates the absence of the gene.

The 7dupASD, also known as Somerville–van der Aa syndrome, is caused by duplication of the same WBS region. Symptoms of patients with 7dupASD have cognitive abnormalities, such as language delay and deficits in social interaction [63,64]. Thus, opposite and graded phenotypes have been observed between patients with WBS and patients with 7dupASD (microdeletion and duplication of WBS region, respectively) (Figure 2) [63,64,65], suggesting that dosage of genes in this region is an important determinant of the symptoms in WBS and 7dupASD. Thus, copy number variations (CNVs) of the WBS region are related to some symptoms of WBS and 7dupASD. This suggests that accuracy of the expression level (dosage) of the genes in this region is critical for normal neuronal development related to symptoms in WBS and 7dupASD. This concept is in good agreement with the notion that CNVs (deletions or duplications of small chromosomal regions) are responsible for various neuropsychiatric disorders [65]. Alteration of Gtf2i expression may alter the expression profile of the downstream genes via its role in transcription regulation. This may be one of the molecular mechanisms underlying CNV effects (regulation by GTF2I dosage) in WBS and 7dupASD.

Induced pluripotent stem cells (iPSCs) prepared from both patients with WBS and patients with 7dupASD demonstrated that dosage of the WBS region contributes to transcriptional dysregulation of genes involved in disease-relevant pathways, such as cell adhesion, migration, calcium homeostasis, inner ear morphogenesis, craniofacial phenotypes, and kidney epithelium development. These processes are deeply related to WBS and 7dupASD [66]. For example, an experiment using four different conditions of GTF2I dosage level produced by lentivirus-mediated RNA interference against GTF2I from this patient-derived iPSCs found that expression of the transcription factor BEN domain containing 4 (BEND4), which is immediately downstream of GTF2I, was repressed in a dosage-dependent manner [66]. This further supports the involvement of GTF2I in WBS and 7dupASD.

### 4.2. GTF2I Abnormality Is Related to Neuropsychiatric Diseases

Some neuropsychiatric disease-related single nucleotide polymorphisms (SNPs) have been found in *GTF2I*. Two SNPs in *GTF2I* related to ASD were found in a study on 1142 individuals with ASD, and were observed to be associated with severe deficiency in social skills and repetitive behavior [67]. These two SNPs of *GTF2I* reside in the intron between the exon 1 (encoding the 5′UTR) and the exon 2 (including the start codon of *GTF2I*). Because this region contains multiple splice sites and produces various alternative 5′UTRs [19,20], these SNPs may cause abnormal splicing of the *GTF2I* 5′UTR (in both human and rat). Intriguingly, from a search in the 5′UTR region of the *GTF2I* genomic sequence using the NCBI SNP database (Available at: https://www.ncbi.nlm.nih.gov/snp/?term=GTF2I), there are more than 10 SNPs in the 5′UTR of *GTF2I*. These 5′UTR SNPs may be related to altered localization and translation of *GTF2I* mRNA. These findings shed light on the importance of 5′UTR splice variation of *GTF2I* for neuronal function.

As previously mentioned, Gtf2i may affect the excitatory/inhibitory balance of brain through *Dlx5*/*Dlx6* regulation. Disruption of this pathway has been suggested to be a possible mechanism that causes autism [68,69]. Indeed, human *DLX5/DLX6* are associated with ASD [70]. Furthermore, autism-associated SNPs have been reported in I56i, an enhancer element of the *DLX*5/*DLX6*. These mutations may alter the binding properties of this element to GTF2I and DLX2, which would impair the transcriptional activation of I56i [47]. Alteration of the excitation/inhibition balance is a hypothetical basis of ASD [68]. Aberrant development of GABAergic interneurons reportedly results in neurodevelopmental disorders, such as epilepsy, schizophrenia, and ASDs, including tuberous sclerosis, fragile X syndrome, Angelman syndrome, and Rett syndrome [69,71]. Thus, Gtf2i abnormality (e.g., abnormal Gtf2i expression level), via imbalance of the excitatory/inhibitory neuronal balance in the brain, may result in the development of ASD symptoms.

## 5. Concluding Remarks

Focusing on Gtf2i, here, we reviewed and discussed the roles of 5′UTRs in mRNA targeting into dendrites and translational regulation within dendrites. In many neuropsychiatric diseases, including WBS, the effects of dosage regulation of genes on the pathogenesis of neuropsychiatric disorders have been recently emphasized. Dosage alteration may impair transcription of downstream genes, which may affect neural development, neural structure, and circuit formation related to neuropsychiatric disorders. We suggest that the 5′UTR of *Gtf2i* mRNA may play a role in the spatiotemporal specific regulation of its protein level via postsynaptic local translation. Direct evidences showing the involvement of 5′UTRs in neuropsychiatric disorders still needs to be established. One powerful method is finding 5′UTR mutation(s) of causal genes related to neuropsychiatric disorders by whole genome sequencing of patient samples. Further studies on dosage regulation of causal genes in neuropsychiatric disorders should be advanced.

## Abbreviations

7dupASD	7q microduplication syndrome
ASD	autism spectrum disorder
BDNF	brain-derived neurotrophic factor
CNV	copy number variation
FMRP	fragile X mental retardation protein
GTF2I	general transcription factor II-I
iPSCs	induced pluripotent stem cells
NLS	nuclear localization signal
PSD	postsynaptic density
RBP	RNA-binding protein
SNP	single nucleotide polymorphisms
TSS	transcription start site
UTR	untranslated region
WBS	Williams–Beuren syndrome
